# Supplementary material for: Identification of Meibomian gland stem cell populations and mechanisms of aging
Source: Nat Commun. 2025 Feb 15;16:1663. doi: 10.1038/s41467-025-56907-6 (PMC11830078; doi:10.1038/s41467-025-56907-6)
Supplement: Supplementary file 1 — Supplementary Information [file 41467_2025_56907_MOESM1_ESM.pdf]

SUPPLEMENTARY DATA

Supplementary Figures S1-S7

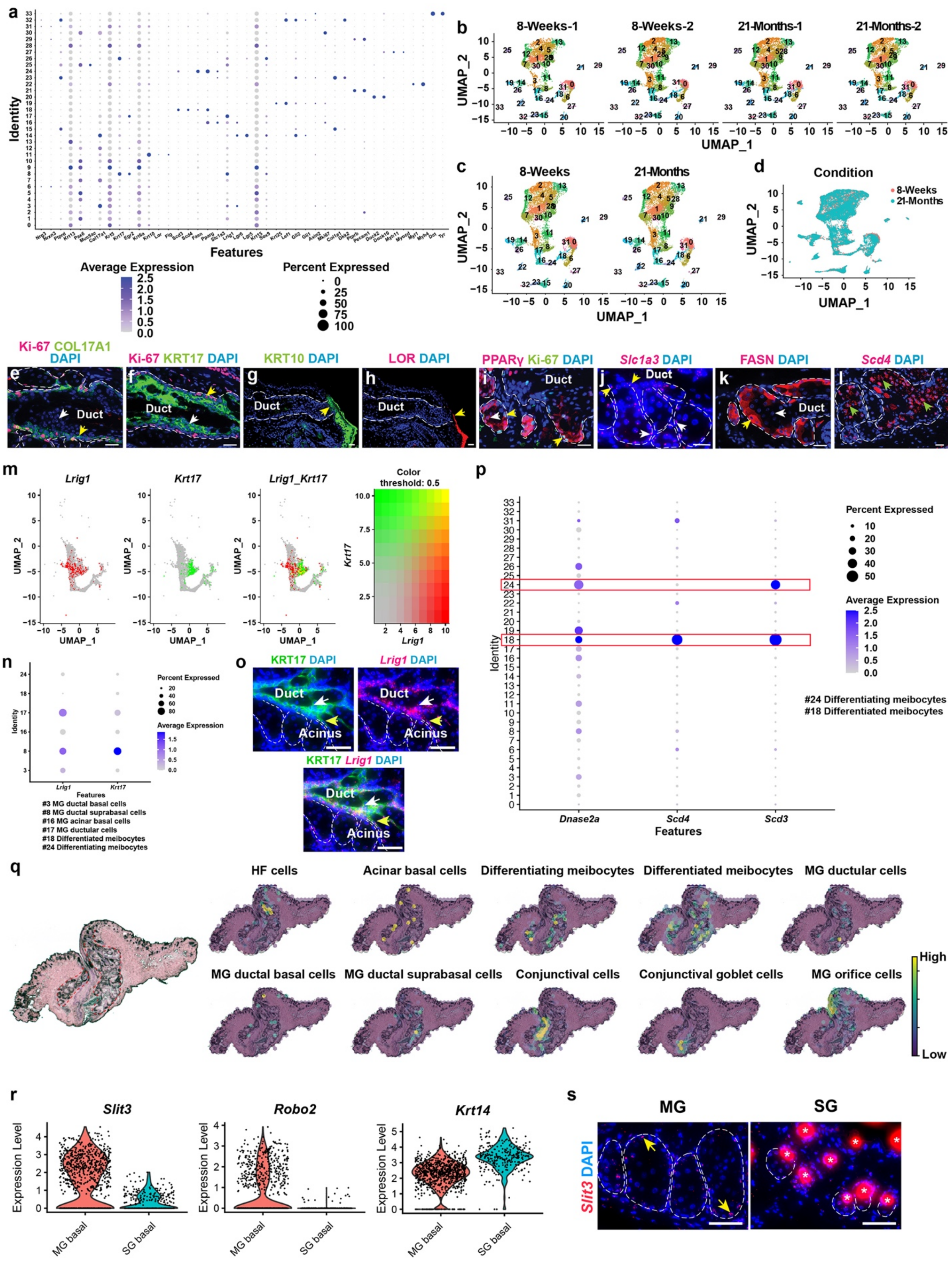

Supplementary Fig. S1. Identification of sub-populations of MG cells in snRNA-seq

datasets and validation of cluster assignment via RNAscope and spatial transcriptomics. (a) Dot plot shows the expression of signature genes for each cell cluster. (b) Separate UMAP plots for the four snRNA-seq datasets. (c) Separate UMAP plots for the combined replicate snRNA-seq datasets from 8-week-old and 21-month-old tarsal plates. (d) Merged UMAP plots of snRNA-seq datasets from 8-week-old and 21-month-old tarsal plates. (e) IF data show expression of COL17A1 in the Ki-67$^+$ basal layer of MG ducts (yellow arrow) and its absence from the MG ductal suprabasal layer (white arrow). (f) IF for KRT17 shows that it is highly expressed in the MG ductal suprabasal layer (white arrow) and is weakly expressed in some ductal basal cells that are positive for Ki67 (yellow arrow). (g) IF shows that KRT10 is expressed in the ductal orifice (yellow arrow). (h) IF shows that LOR is expressed in the ductal orifice suprabasal layer (yellow arrow). (i) IF shows that PPARγ is expressed in Ki-67$^+$ proliferative acinar basal cells (yellow arrows) and differentiating meibocytes (white arrow). (j) RNAscope for *Slc1a3* shows that it is primarily expressed in MG acinar basal cells (white arrows) and ductular cells (yellow arrows). (k) IF for FASN shows that it is expressed in differentiating meibocytes (yellow arrow) and not in fully differentiated meibocytes (white arrow). (l) RNAscope for *Scd4* shows that it is predominantly expressed in differentiated meibocytes (green arrows). (m) Feature plots showing expression of *Lrig1* and *Krt17* in MG cells. The yellow color represents overlapping expression. (n) Dot plot indicating expression of *Lrig1* and *Krt17* in MG cells. (o) IF for KRT17 and RNAscope for *Lrig1* showing their overlapping expression in MG ducts (white arrows) and ductules (yellow arrows). (p) Dot plot showing the expression of *Dnase2a*, *Scd3* and *Scd4* in MG cells. *Scd3* and *Scd4* are expressed in differentiating and differentiated meibocytes more specifically than *Dnase2a* which also localizes to other MG subpopulations. (q) Spatial transcriptomics analysis of 8-week MG using the 10x Visium platform; the results were integrated with annotations based on the snRNA-seq data. (r) Violin plots comparing expression of *Slit3*, *Robo2* and *Krt14* in MG acinar basal cells versus hair follicle-associated SG basal cells. SG RNA-seq data were obtained from dataset GSE225252[16] in the GEO database. *Slit3* and *Robo2* showed higher expression in MG compared with SG basal cells, while *Krt14* was expressed more highly in SG than MG basal cells. (s) RNAscope for *Slit3* shows its expression is higher in MG acinar basal cells than SG basal cells. White asterisks indicate autofluorescence from hair shafts. White dashed lines in (e-l, o, s) outline the MG duct and/or acinus. Scale bars in (e-l, o) represent 25 μm; scale bars in (s) represent 50 μm. N=3 independent samples from 2 male C57BL/6J mice and 1 female C57BL/6J mouse at 8 weeks of age were analyzed for IF and RNAscope. Representative images are shown. Related to Fig. 1.

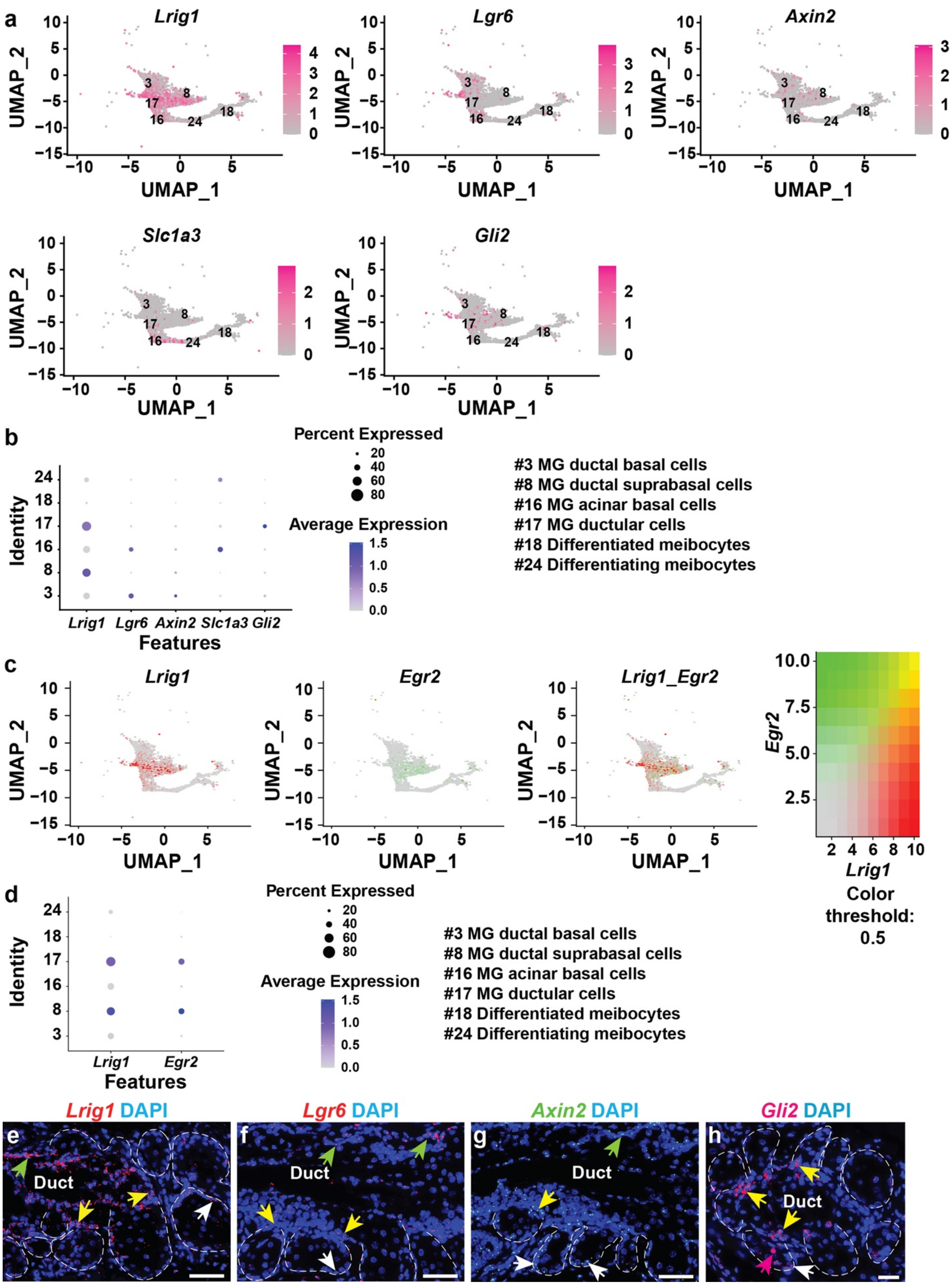

Supplementary Fig. S2. Expression of stem cell markers in the MG. (a, b) Feature plots (a)

and dot plot (b) from snRNA-seq data showing expression of *Lrig1*, *Lgr6*, *Axin2*, *Slc1a3*, and *Gli2* in MG cell populations. (c) Feature plots reveal partially overlapping expression of *Lrig1* and *Egr2* in MG cell populations. (d) Dot plot of snRNA-seq data shows enrichment of *Lrig1* and *Egr2* expression in MG duct suprabasal cells and ductular cells. (e) RNAscope validates that *Lrig1* is highly expressed in central duct (green arrow), moderately expressed in ductule (yellow arrows), and weakly expressed in the acinar basal layer. (f) RNAscope for *Lgr6* shows that it is broadly expressed in MG central duct (green arrows), ductule (yellow arrows), and acinar basal layer (white arrow). (g) RNAscope for *Axin2* shows that it is broadly expressed in central duct (green arrow), ductule (yellow arrow), and acinar basal layer (white arrows). (h) RNAscope for *Gli2* shows it is enriched in ductule (yellow arrows) and is also detected in the acinar basal layer (white arrow) and a sub-population of meibocytes (pink arrow). White dashed lines in (e-h) outline the MG acini. n=3 independent samples from 2 male C57BL/6J mice and 1 female C57BL/6J mouse at 8 weeks of age were used for RNAscope. Representative images are shown. Scale bars: (e-g), 50μm; (h), 25μm. Related to Fig. 2.

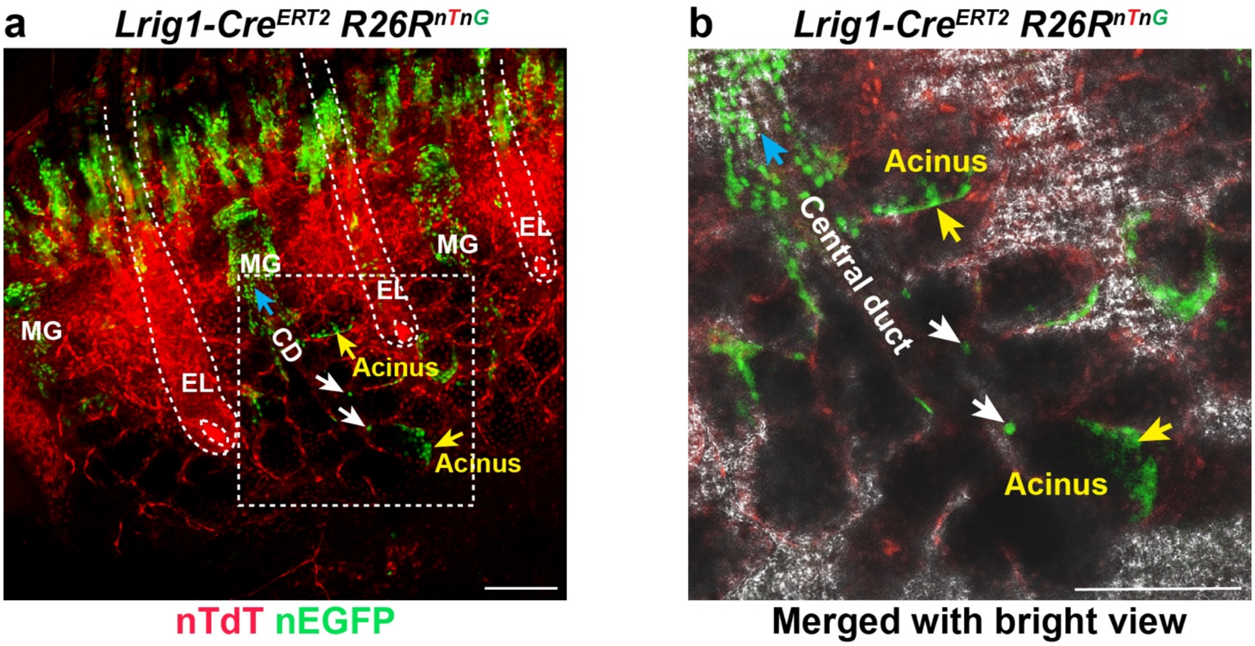

Supplementary Fig. S3. Progeny of *Lrig1*$^{+}$ stem cells are present in MG ductules, acini and central ducts. (a) Whole mount confocal image of an MG explant from an *Lrig1-Cre*^{ERT2} *R26R*^{nTnG} mouse induced at P36 and analyzed at P76. Lrig1 lineage traced cells (green) are present in ductules (white arrows), acini (yellow arrows) and central duct (blue arrow). (b) Enlarged view of the region outlined by a dashed white line in panel (a) and merged with bright view image. Grey dashed lines in (a) outline the eyelash follicles. n=2 (1 male and 1 female) mice were analyzed. Representative images are shown. EL, eyelash follicle; MG, Meibomian gland; CD, central duct. Scale bars: 150 μm. Related to Fig. 2.

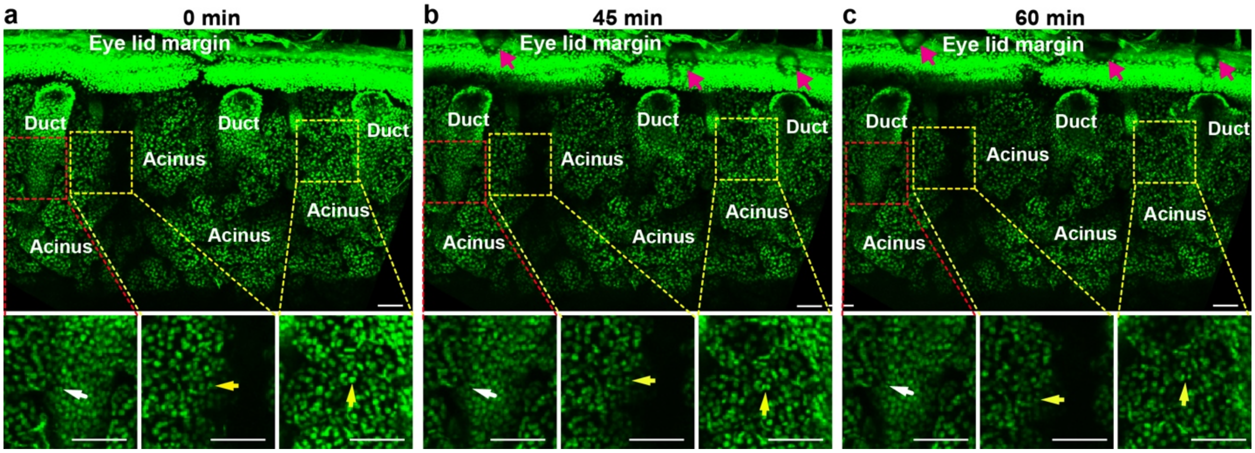

Supplementary Fig. S4. MGs in tarsal plate explants undergo proliferation and secrete lipid droplets during short-term culture. (a-c) Representative stills taken at 0 minutes (a), 45 minutes (b) and 60 minutes (c) from time-lapse live imaging of an adult *K14:H2B-GFP*[74] murine MG explant in which all MG cells are GFP^+. Areas shown by colored dashed boxes are shown at higher magnification beneath each image. Pink arrows indicate extruded drops of meibum. White arrows indicate a dividing cell in the central duct; yellow arrows indicate dividing cells in the acini. n=2 samples from 1 male and 1 female mouse at 8 weeks of age were analyzed. Scale bars: 100µm. Related to Fig. 2.

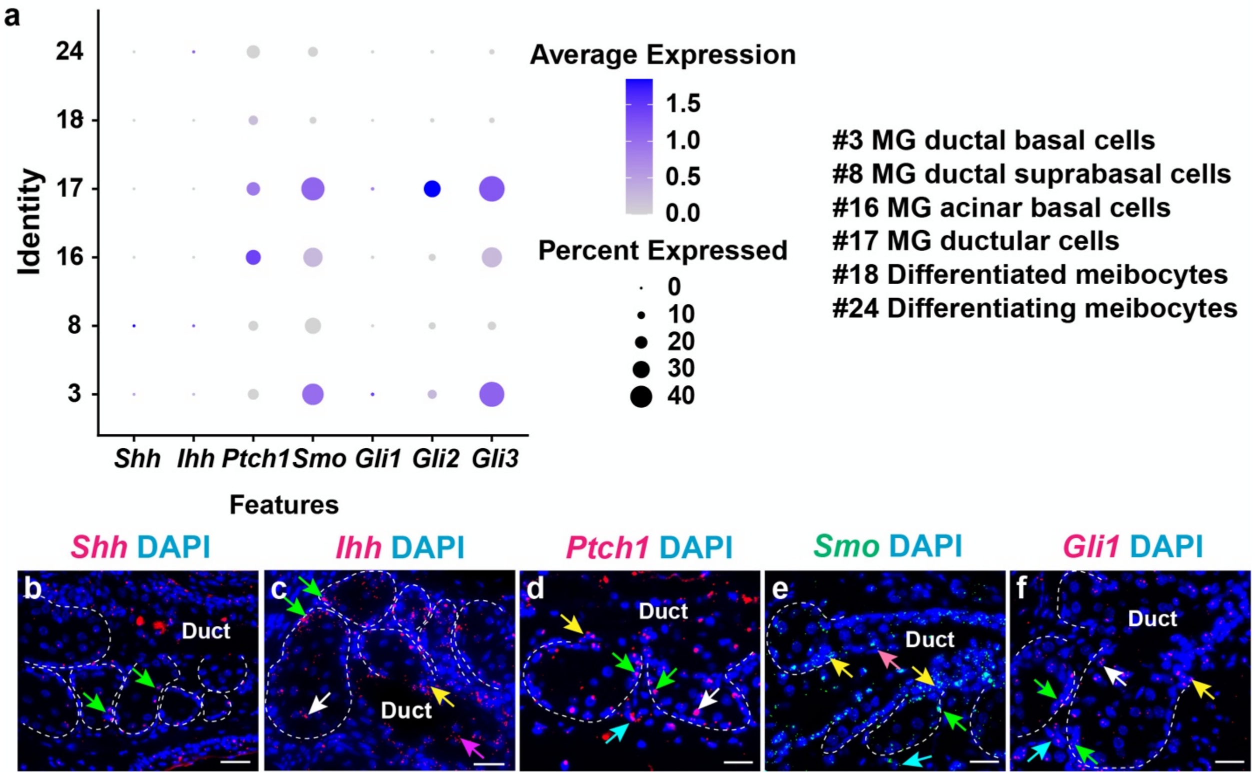

Supplementary Fig. S5. Hh pathway components are expressed in the MG. (a) Dot plot of snRNA-seq data shows that Hh pathway components are expressed in MG subpopulations. (b-f) RNAscope (red) reveals expression of *Shh* in acinar basal cells (b, green arrows); *Ihh* in acinar basal cells (c, green arrows), differentiating meibocytes (c, white arrow), ductular cells (c, yellow arrow), and ductal suprabasal cells (c, purple arrow); *Ptch1* in acinar basal cells (d, green arrows), differentiating meibocytes (d, white arrow), ductular cells (d, yellow arrow), and stromal cells (d, light blue arrow); *Smo* expression in acinar basal cells (e, green arrow), ductular cells (e, yellow arrows), ductal basal cells (e, pink arrow), and stromal cells (e, light blue arrow); and *Gli1* in acinar basal cells (f, green arrows), differentiating meibocytes (f, white arrow), ductular cells (f, yellow arrow), and stromal cells (f, light blue arrow). White dashed lines in (b-f) outline acini. For RNAscope, n=3 C57BL/6J mice (2 males and 1 female) were analyzed at 8 weeks of age. Representative images are shown. Scale bars: 25 µm. Related to Fig. 3.

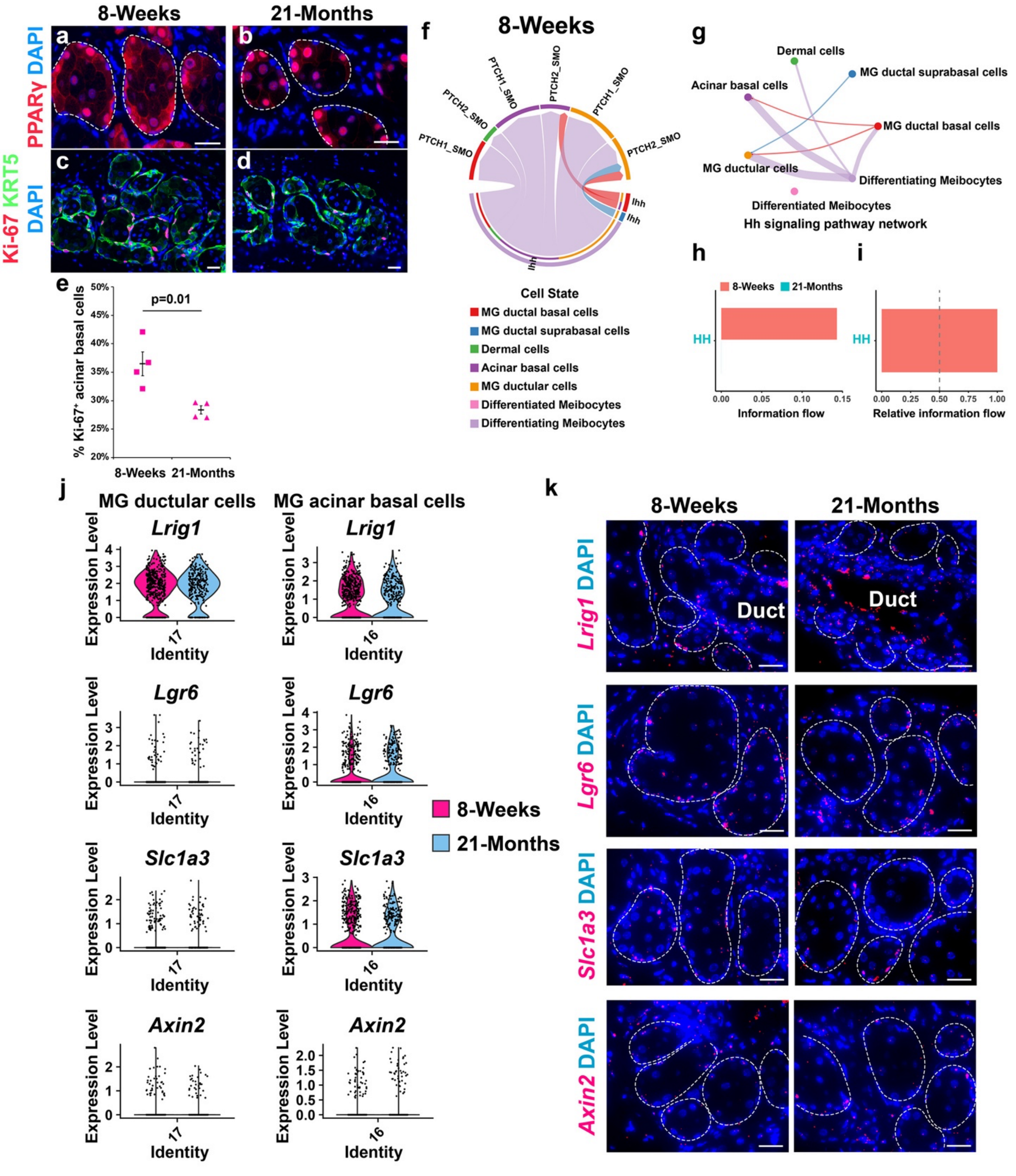

Supplementary Fig. S6. Decreased proliferation and Hh signaling in aged MGs. (a, b) IF data show that PPARγ is detected in the cytoplasm and nucleus of acinar cells in young MG (a) but is lost from the cytoplasm and enriched in the nucleus of acinar cells in aged MG (b). (c, d) Ki-67/KRT5^{+} proliferating acinar basal cells are reduced in aged (d) compared with young (c) MG. (e) Quantification shows that the percentage of KRT5^{+} basal cells that are Ki-67^{+} is statistically significantly reduced in aged compared with young MGs. n=4 male C57BL/6J mice were analyzed per condition in (a-e). At least 114 acinar basal cells were analyzed per animal in (e). Statistical significance was calculated using unpaired two-tailed Student's t-test. Data are

presented as mean +/- SEM. Source Data are provided as a Source Data file. (f, g) CellChat analysis indicating predicted Hh pathway-mediated intercellular communications among the indicated cell populations in young MG cells and between MG epithelial populations and surrounding dermal cells. Notably, no significant Hh pathway interactions were identified within aged MG cells or between aged MGs and surrounding dermal cells, due to extremely low Hh pathway gene expression in aged MGs. (h, i) CellChat analysis data depicting predicted intercellular crosstalk within MGs and between MGs and surrounding dermal cells mediated by Hh signaling pathways (h) and its relative strength in young and aged MGs (i). (j, k) Violin plots (j) and RNAscope data (k) show similar expression levels of *Lrig1*, *Lgr6*, *Slc1a3*, and *Axin2* in young and aged MGs. Samples from n=4 8-week and n=4 21-month-old male C57BL6/J mice were analyzed in (k); representative data are shown. White dashed lines in (a, b, k) outline MG acini. Scale bars represent 25 µm. Related to Fig. 7.

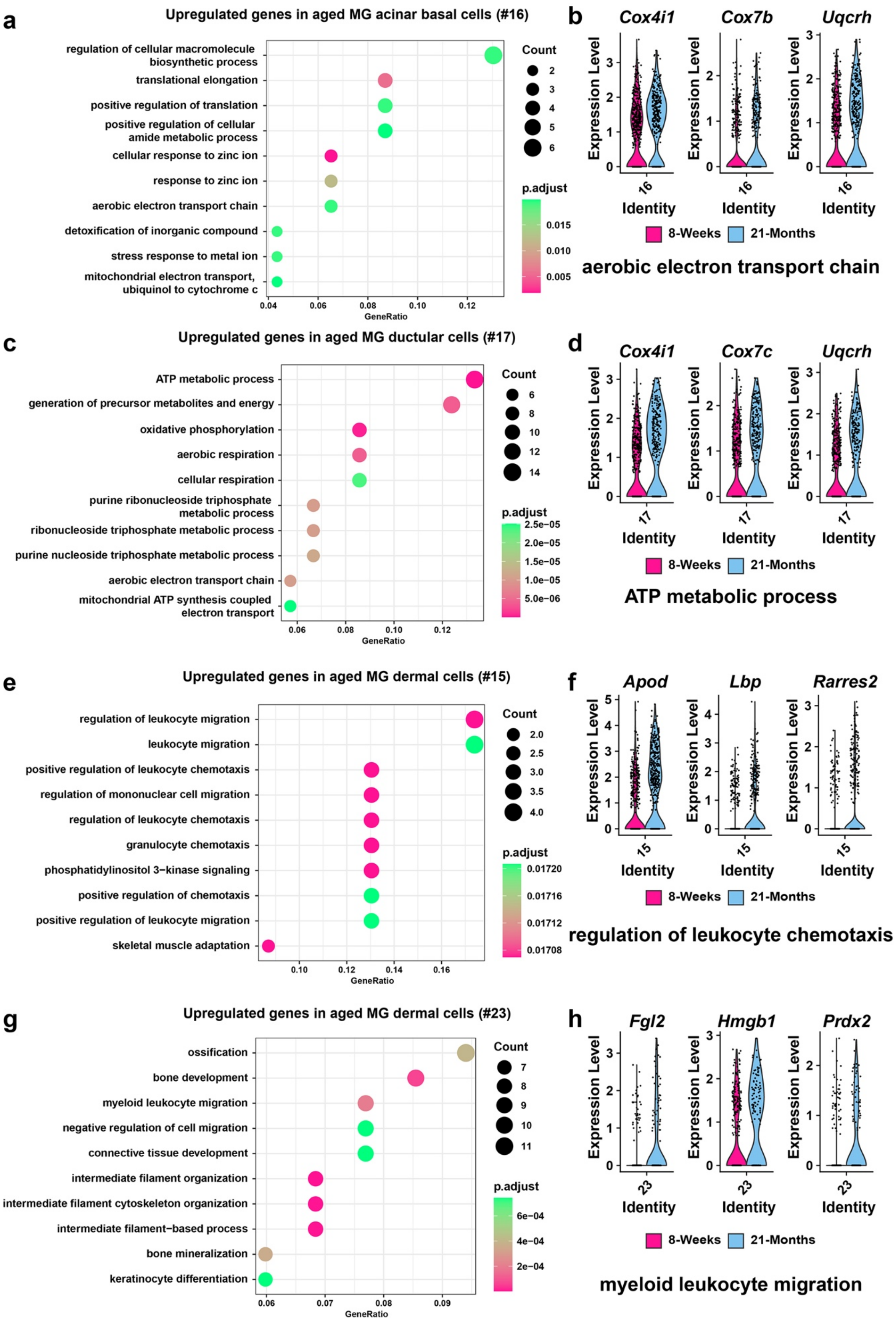
a
Upregulated genes in aged MG acinar basal cells (#16)
regulation of cellular macromolecule biosynthetic process
translational elongation
positive regulation of translation
positive regulation of cellular amide metabolic process
cellular response to zinc ion
response to zinc ion
aerobic electron transport chain
detoxification of inorganic compound
stress response to metal ion
mitochondrial electron transport, ubiquinol to cytochrome c
GeneRatio
Count
p.adjust
b
Cox4i1
Cox7b
Uqcrh
Expression Level
Identity
8-Weeks
21-Months
aerobic electron transport chain
c
Upregulated genes in aged MG ductular cells (#17)
ATP metabolic process
generation of precursor metabolites and energy
oxidative phosphorylation
aerobic respiration
cellular respiration
purine ribonucleoside triphosphate metabolic process
ribonucleoside triphosphate metabolic process
purine nucleoside triphosphate metabolic process
aerobic electron transport chain
mitochondrial ATP synthesis coupled electron transport
d
Cox4i1
Cox7c
Uqcrh
ATP metabolic process
e
Upregulated genes in aged MG dermal cells (#15)
regulation of leukocyte migration
leukocyte migration
positive regulation of leukocyte chemotaxis
regulation of mononuclear cell migration
regulation of leukocyte chemotaxis
granulocyte chemotaxis
phosphatidylinositol 3−kinase signaling
positive regulation of chemotaxis
positive regulation of leukocyte migration
skeletal muscle adaptation
f
Apod
Lbp
Rarres2
regulation of leukocyte chemotaxis
g
Upregulated genes in aged MG dermal cells (#23)
ossification
bone development
myeloid leukocyte migration
negative regulation of cell migration
connective tissue development
intermediate filament organization
intermediate filament cytoskeleton organization
intermediate filament−based process
bone mineralization
keratinocyte differentiation
h
Fgl2
Hmgb1
Prdx2
myeloid leukocyte migration

Supplementary Fig. S7. GO analysis of upregulated genes in aged MG cells and surrounding dermal cells. (a, b) GO analysis of genes with increased expression in aged compared with young acinar basal cells (cluster #16) (a) and violin plots for aerobic electron transport chain genes in acinar basal cells (b). (c, d) GO analysis of genes with increased expression in aged compared with young MG ductular cells (cluster #17) (c) and violin plots for oxidative phosphorylation pathway genes in ductular cells (d). (e, f) GO analysis of genes with increased expression in aged compared with young cluster #15 dermal cells (e) and violin plots for leukocyte chemotaxis genes in cluster #15 dermal cells (f). (g, h) GO analysis of genes with increased expression in aged compared with young cluster #23 dermal cells (g) and violin plots for myeloid leukocyte migration genes in cluster #23 dermal cells (h). FDR calculation in (a, c, e, g) was performed by clusterProfiler (v4.4.4) and EdgeR (v3.38.4) with the Benjamini-Hochberg procedure. Dot plots in (a, c, e, g) were generated with ggplot2. Violin plots in (b, d, f, h) were generated with the VlnPlot command from Seurat package. Related to Fig. 9.
