# Supplementary material for: Identification of Meibomian gland stem cell populations and mechanisms of aging
Source: Nat Commun. 2025 Feb 15;16:1663. doi: 10.1038/s41467-025-56907-6 (PMC11830078; doi:10.1038/s41467-025-56907-6)
Supplement: Supplementary file 3 — Description of Additional Supplementary Files [file 41467_2025_56907_MOESM3_ESM.pdf]

<u>**Description of Additional Supplementary Files**</u>

Supplementary Movie 1. MGs in tarsal plate explants undergo proliferation and secrete lipid droplets during short-term culture. Time-lapse confocal live imaging of 8-week K14:H2B-GFP murine MG explant in which all MG cells are GFP+. Note extrusion of droplets of meibum. Red arrow indicates a dividing cell in the central duct; yellow arrows indicate dividing cells in the acini. Samples from n=2 mice were analyzed (1 male and 1 female). Related to Figure 1.

Supplementary Movie 2. Lrig1+ MG ductule stem cell progeny can migrate toward acinus and locate in the acinus basal layer. Time-lapse confocal imaging over 15 hours of culture of an MG explant from an Lrig1-CreERT2 ROSAnTnG male mouse induced from P40-P42 and analyzed at P102. The white arrowhead labels an Lrig1+ stem cell migrating from the ductule towards the acinus. The red arrowhead marks a ductular Lrig1+ stem cell that divides once and then both daughter cells, indicated respectively with green and yellow arrowheads, migrate toward the acinus. The central duct is outlined with white dots; the acini are outlined with red dashed lines. Samples from n=2 mice were analyzed. Related to Figure 1.
