# Supplementary material for: Identification of Meibomian gland stem cell populations and mechanisms of aging
Source: Nat Commun. 2025 Feb 15;16:1663. doi: 10.1038/s41467-025-56907-6 (PMC11830078; doi:10.1038/s41467-025-56907-6)
Supplement: Supplementary file 6 — Reporting Summary [file 41467_2025_56907_MOESM6_ESM.pdf]

nature portfolio

Corresponding author(s): Carlo Iomini and Sarah E. Millar

Last updated by author(s): 2025-22-01

# Reporting Summary

Nature Portfolio wishes to improve the reproducibility of the work that we publish. This form provides structure for consistency and transparency in reporting. For further information on Nature Portfolio policies, see our Editorial Policies and the Editorial Policy Checklist.

## Statistics

For all statistical analyses, confirm that the following items are present in the figure legend, table legend, main text, or Methods section.

| n/a | Confirmed | |
|---|---|---|
| ☐ | ☒ | The exact sample size (*n*) for each experimental group/condition, given as a discrete number and unit of measurement |
| ☐ | ☒ | A statement on whether measurements were taken from distinct samples or whether the same sample was measured repeatedly |
| ☐ | ☒ | The statistical test(s) used AND whether they are one- or two-sided<br>*Only common tests should be described solely by name; describe more complex techniques in the Methods section.* |
| ☒ | ☐ | A description of all covariates tested |
| ☒ | ☐ | A description of any assumptions or corrections, such as tests of normality and adjustment for multiple comparisons |
| ☐ | ☒ | A full description of the statistical parameters including central tendency (e.g. means) or other basic estimates (e.g. regression coefficient) AND variation (e.g. standard deviation) or associated estimates of uncertainty (e.g. confidence intervals) |
| ☐ | ☒ | For null hypothesis testing, the test statistic (e.g. *F*, *t*, *r*) with confidence intervals, effect sizes, degrees of freedom and *P* value noted<br>*Give P values as exact values whenever suitable.* |
| ☒ | ☐ | For Bayesian analysis, information on the choice of priors and Markov chain Monte Carlo settings |
| ☒ | ☐ | For hierarchical and complex designs, identification of the appropriate level for tests and full reporting of outcomes |
| ☒ | ☐ | Estimates of effect sizes (e.g. Cohen's *d*, Pearson's *r*), indicating how they were calculated |

*Our web collection on statistics for biologists contains articles on many of the points above.*

## Software and code

Policy information about availability of computer code

| | |
|---|---|
| Data collection | No software was used for data collection. |
| Data analysis | Sample size for experiments involving quantitation was pre-determined using the statsmodels (0.9.0) package in Python (3.7)<br>Statistical analysis and graphical representation were performed using Microsoft Excel 2023.<br>Cell Ranger Single-Cell Software Suite (v7.0.1, 10x Genomics) was used to align and quantify sequencing data against the mm10 mouse reference genome (refdata-gex-mm10-2020-A).<br>Cell clustering and differential gene expression were analyzed using Seurat70 (v4.3.0).<br>For RNA velocity and pseudotime analyses, bam files generated from Cell Ranger were processed using Velocyto (v0.6)'s run10x function with default parameters , resulting in loom files that contained counts of unspliced and spliced mRNAs. The loom files were merged with the h5ad files, which covered the meibomian gland clusters. Subsequently, the merged files were preprocessed with scVelo (v0.3), and RNA velocity and pseudotime analyses were performed using cellDancer (v1.1).<br>To identify DEGs, genes encoding ribosomal proteins were initially defined and removed by grepping for “^Rp(sl)” from the datasets before pseudobulk analysis using EdgeR (v3.38.4). Genes with an FDR (padj)<0.05 and \|Log2FC\|>0.5 were considered DEGs and were subjected to gene ontology analysis with clusterProfiler (v4.4.4).<br>The interactions among distinct MG cell types (MG ductal basal cells, ductal suprabasal cells, ductular cells, acinar basal cells, differentiating meibocytes, and differentiated meibocytes), along with surrounding dermal cells (two populations were combined in this context), were analyzed using CellChat (v1.6.1).<br>Spatial transcriptomics sequencing data were aligned and quantified using Space Ranger Software Suite (v2.1.0, 10x Genomics) against the reference genome.<br>Integration of snRNA-seq data and spatial transcriptomic data was conducted through CytoSPACE (v1.0.5)<br>For live imaging, images were maximum projected with Leica LAS X 3.6.0.20104 software to generate the time-lapse video and video |

annotations were performed with Fiji software 2.1.0.
For bulk RNA-seq data analysis, Salmon (v0.9.1) was used to count data against the transcriptome defined in Gencode vM18. We then annotated and summarized the transcriptome count data to the gene level with tximeta and performed further annotation with biomaRt (v0.7.0). Normalizations and statistical analyses were carried out with DESeq2 (v1.20.0). Genes with padj<0.001 and |Log2FC|>0.5 were selected for GO enrichment analysis, which was carried out by clusterProfiler. The results were plotted by ggplot2 (v3.4.0) and volcano plots were generated using EnhancedVolcano (v1.14.0).

For manuscripts utilizing custom algorithms or software that are central to the research but not yet described in published literature, software must be made available to editors and reviewers. We strongly encourage code deposition in a community repository (e.g. GitHub). See the Nature Portfolio guidelines for submitting code & software for further information.

## Data

Policy information about availability of data

All manuscripts must include a data availability statement. This statement should provide the following information, where applicable:

- Accession codes, unique identifiers, or web links for publicly available datasets
- A description of any restrictions on data availability
- For clinical datasets or third party data, please ensure that the statement adheres to our policy

The authors declare that the main data supporting the findings of this study are available within the article and its Supplemental Information files. Source data are provided with this paper. This study did not generate unique new reagents. snRNA-seq data generated in this study have been deposited in the GEO database under accession code  GSE274498 "snRNA-seq of murine tarsal plates". The spatial transcriptomics data generated in this study have been deposited in the GEO database under accession code GSE274497 "Spatial transcriptomics of murine eyelid". Bulk RNA-seq data generated in this study have been deposited in the GEO database under accession code GSE274496 "Bulk RNA-sequencing of Meibomian glands from Krt5-rtTA tetO-GLI2DN mice and littermate controls". Further information and requests for resources and reagents or for the minimum dataset necessary to interpret, verify and extend the research in the article, should be directed to and will be fulfilled by Sarah E. Millar (sarah.millar@mssm.edu). No restrictions are placed on access to the minimum dataset.

## Research involving human participants, their data, or biological material

Policy information about studies with human participants or human data. See also policy information about sex, gender (identity/presentation), and sexual orientation and race, ethnicity and racism.

| | |
|---|---|
| Reporting on sex and gender | Human samples were provided to the investigators as de-identified samples that had been discarded from unrelated clinical procedures. The investigators did not have access to information on sex, gender, sexual orientation, race or ethnicity. As such, this is not considered as human subjects research. |
| Reporting on race, ethnicity, or other socially relevant groupings | Human samples were provided to the investigators as de-identified samples that had been discarded from unrelated clinical procedures. The investigators did not have access to information on sex, gender, sexual orientation, race or ethnicity. As such, this is not considered as human subjects research. |
| Population characteristics | See above. |
| Recruitment | Not applicable. The study did not involve recruitment of human subjects. |
| Ethics oversight | Use of the human tissue samples in research was approved by the University of Pennsylvania IRB under protocol #827926, PI Vivian Lee. |

Note that full information on the approval of the study protocol must also be provided in the manuscript.

# Field-specific reporting

Please select the one below that is the best fit for your research. If you are not sure, read the appropriate sections before making your selection.

☒ Life sciences ☐ Behavioural & social sciences ☐ Ecological, evolutionary & environmental sciences

For a reference copy of the document with all sections, see nature.com/documents/nr-reporting-summary-flat.pdf

# Life sciences study design

All studies must disclose on these points even when the disclosure is negative.

| | |
|---|---|
| Sample size | Sample size for experiments involving quantitation was pre-determined using the statsmodels (0.9.0) package in Python (3.7). Experiments involving quantification of data used n=5 or n-=4 mice per group. N=5 mice per group provides 80% power at a two-sided significance level of 0.05 to detect a difference (effect size) of 2.0s where s is the standard deviation. n=4 mice per group provides 66% power at a two-sided significance level of 0.05 to detect a difference (effect size) of 2.0s. At least 3 independent biological samples were used for semi-quantitative experiments involving RNAscope and immunostaining; this is a sufficient sample size to ensure reproducibility  of results, based on prior experience. |
| Data exclusions | Nuclei that did not meet the criteria for high quality (defined as nFeature_RNA >200 & nFeature_RNA <4500 & nCount_RNA >500 & < nCount_RNA <25000 & precent.mt < 5) were excluded from the analysis of snRNA-seq data. |

| | |
|---|---|
| Replication | snRNA-seq experiments used 2 independent biological replicates per stage, each pooled from 4 animals; live imaging studies each used 2 independent biological replicates; all other studies used at least 3 independent biological replicates. |
| Randomization | Not applicable to this study: experimental animals were assigned to groups based on genotype or age |
| Blinding | Samples were analyzed in a blinded fashion with respect to genotype, sex, and/or age |

# Reporting for specific materials, systems and methods

We require information from authors about some types of materials, experimental systems and methods used in many studies. Here, indicate whether each material, system or method listed is relevant to your study. If you are not sure if a list item applies to your research, read the appropriate section before selecting a response.

## Materials & experimental systems

| n/a | Involved in the study | |
|---|---|---|
| ☐ | ☒ | Antibodies |
| ☒ | ☐ | Eukaryotic cell lines |
| ☒ | ☐ | Palaeontology and archaeology |
| ☐ | ☒ | Animals and other organisms |
| ☒ | ☐ | Clinical data |
| ☒ | ☐ | Dual use research of concern |
| ☒ | ☐ | Plants |

## Methods

| n/a | Involved in the study | |
|---|---|---|
| ☒ | ☐ | ChIP-seq |
| ☒ | ☐ | Flow cytometry |
| ☒ | ☐ | MRI-based neuroimaging |

## Antibodies

| | |
|---|---|
| Antibodies used | Rat monoclonal anti-Ki-67 (eBioscience #13-5689-82; 1:200); Rabbit anti-COL17A1 (Abcam #ab184996, 1:500, RRID AB_3073438); Rabbit anti-KRT17 (Abcam # ab109725, 1:200, RRID AB_10889888); Mouse anti-KRT10 (Novus #NBP2-47650, 1:200); Rabbit anti-LOR (BioLegend #905103, 1:500, RRID AB_2734676); Rabbit anti-PPARγ (Cell Signaling Technology #2435T, 1:200, RRID AB_2166051); Rabbit anti-FASN (Cell Signaling Technology #3180T, 1:200, RRID AB_2100796); Guinea Pig anti-PLIN2 (Fitzgerald #20R-AP002, 1:500, AB_1282475); Rabbit anti-PLIN2 (Sigma-Aldrich #393A-1, 1:100); Rabbit anti-GFP (Abcam #ab290, 1:1000, RRID AB_2313768); Rabbit anti-KRT5 (BioLegend #905504, 1:500, RRID AB_2616956); Rabbit anti-KRT14 (Thermo Fisher Scientific #MA5-11599, 1:400, RRID AB_10982092); Rabbit anti-ERK1/2 (Cell Signaling Technology #4695T, 1:100, RRID AB_390779); Rabbit anti-p-ERK1/2 (Cell Signaling Technology #4370T, 1:100, RRID AB_2315112); Mouse anti-Myctag (Cell Signaling Technology #2276S, 1:1000, RRID AB_331783); Rabbit anti-GLI2 (Novus Biologicals #NB600-874SS, 1:200, RRID AB_10001953); Goat anti-GLI2 (R&D Systems #AF-3635, 1:50, RRID AB_2111902); Rabbit anti-Acetylated-Lysine (Cell Signaling Technology #9441s, 1:100, RRID AB_331805); Rabbit anti-HDAC1 (Thermo Fisher Scientific #49-1025, 1:1000, RRID AB_2533875); Rabbit anti-HDAC2 (Thermo Fisher Scientific #51-5100, 1:1000, RRID AB_2533908); Donkey anti-Rabbit IgG Secondary Antibody, Alexa Fluor 555 (Invitrogen #A-31572, 1:800, RRID AB_162543); Donkey anti-Mouse IgG Secondary Antibody, Alexa Fluor 488 (Invitrogen #A-21202, 1:800, RRID AB_162543); Goat anti-Guinea Pig, Biotinylated (Vector Laboratories #A-7000, 1:500, RRID AB_2336132); Goat anti-Rabbit, Biotinylated (Vector Laboratories #BA-1000, 1:500, RRID AB_2313606); Streptavidin Fluorescein (Vector Laboratories #SA-5001, 1:500, RRID AB_2336462); Streptavidin Texas Red (Vector Laboratories #SA-5006, 1:500, RRID AB_2336754). |
| Validation | All antibodies had been used successfully in published studies. We additionally tested some antibodies for specificity by IF using knockout mouse tissues. |

## Animals and other research organisms

Policy information about studies involving animals; ARRIVE guidelines recommended for reporting animal research, and Sex and Gender in Research

| | |
|---|---|
| Laboratory animals | C57BL/6J mice (Jackson Laboratory #000664) were used for snRNA-seq, validation of snRNA-seq data via RNAscope and IF, spatial transcriptomics, and aging-related experiments. All other mice were maintained on a mixed C57BL/6J / SJL/J FVB/NJ strain background. The following mouse lines were utilized: Lrig1-CreERT2 (Jackson Laboratory, #018418), Lgr6-CreERT2 (Jackson Laboratory, #016934), Axin2-CreERT2 ref 33, Slc1a3-CreERT2 (European Mouse Mutant Archive, EM:12216), Gli2-CreERT2 ref 67, Krt5-rtTA (Jackson Laboratory, #017519), tetO-GLI2ΔN38, Rosa26mTmG (Jackson Laboratory, #007676), Rosa26nTnG (Jackson Laboratory, #023537), KRT14:H2BGFP ref 68, KRT14-CreERT2 (Jackson Laboratory, #005107), KRT14-CreER (Jackson Laboratory #005107), Smofl/fl (Jackson Laboratory, #004526), Hdac1fl/fl Hdac2fl/fl ref 69, tetO-Cre (Jackson Laboratory #006234), Ptch1fl/fl (Jackson Laboratory #012457).<br><br>Figure 1. snRNA-seq was performed on eight 8-week-old and eight 21-month-old male C57BL/6J mice.<br><br>Figure 2. Lineage tracing experiments used Lrig1-CreERT2, Lgr6-CreERT2, Axin2-CreERT2, Gli2-CreERT2 and Slc1a3-CreERT2 mice carrying a Rosa26mTmG or Rosa26nTnG reporter allele. Mice were tamoxifen treated at P50 and analyzed at P52 and P140 (Lgr6-CreERT2, Gli2-CreERT2 and Slc1a3-CreERT2) or P170 (Lrig1-CreERT2 and Axin2-CreERT2). N=3 mice (2 males and 1 female) of each genotype were analyzed in lineage tracing experiments for each line and stage. |

Figure 3. KRT14-CreERT2 Rosa26mTmG control mice were analyzed 18 or 29 weeks after tamoxifen treatment at 8 weeks of age (T18 Weeks and T29 Weeks) (n=4, 2 male and 2 female); KRT14-CreERT2 Smofl/fl Rosa26mTmG mice were analyzed at T18 Weeks or T29 Weeks (n=4, 3 male and 1 female). n=4 KRT14-CreERT2 control mice (3 male and 1 female) and n=4 Smo-deficient KRT14-CreERT2 Smofl/fl mice (3 male and 1 female) were analyzed by immunostaining at 34 weeks after tamoxifen treatment.

Figure 4. (a-l) n=3 (2 males and 1 female) Krt5-rtTA tetO-GLI2ΔN mice were analyzed after 2, 4 and 7 days of doxycycline treatment starting at 8 weeks of age. N=3 control mice lacking Krt5-rtTA or tetO-GLI2ΔN (2 males and 1 female) and n=3 Gli2  NKrt5-rtTA mice (2 males and 1 female) were analyzed were analyzed after 2, 4 and 7 days of doxycycline treatment starting at 8 weeks of age. (m-p) Samples from n=5 control mice (3 males and 2 females) lacking Krt5-rtTA or tetO-GLI2ΔN and samples from n=5 Krt5-rtTA tetO-GLI2ΔN mice (3 males and 2 females) were analyzed. (s-v) n=3 male KRT14-CreERT2 Ptch1fl/fl mice, n=2 age-matched male controls and n=2 age-matched female controls were analyzed 12 weeks after tamoxifen treatment at 8 weeks of age.

Figure 5. n=3 Krt5-rtTA tetO-GLI2ΔN mice (2 male and 1 female) and n=3 littermate control mice lacking Krt5-rtTA or tetO-GLI2ΔN (2 males and 1 female) were analyzed by bulk RNA-seq 4 days after doxycycline treatment at 8 weeks. 3 Gli2ΔNKrt5-rtTA mice (2 males and 1 female) and 3 littermate controls of genotypes tetO-GLI2ΔN or Krt5-rtTA (2 males and 1 female) were used for RNAscope and IF after doxycycline treatment for 4 days starting at 8 weeks of age.

Lrig1-CreERT2, Lgr6-CreERT2, Axin2-CreERT2, Gli2-CreERT2 and Slc1a3-CreERT2 mice carrying Rosa26mTmG and Krt5-rtTA tetO-GLI2ΔN were tamoxifen induced at P42 to induce Cre activity and placed on oral doxycycline at P72 to induce GLI2ΔN expression. Mice were analyzed by IF at P74 or P82. n=3 (1 male and 2 females) samples were analyzed per line per time point.

Figure 7. (b-I; o-r) 3 male 8-week and 3 male 21-month C57BL/6J mice were analyzed by RNAscope, PLA and IF. (j-m) 3 male Krt5-rtTA tetO-Cre Hdac1fl/fl Hdac2fl/fl experimental mice and 3 male control mice of genotype Hdac1fl/fl Hdac2fl/fl and lacking Krt5-rtTA or tetO-Cre were doxycycline induced from P48 and analyzed at P58 by PLA and RNAscope.

Figure 8. (e) Samples from n=3 8-week and n=3 21-month-old male C57BL/6J mice were used for RNAscope and IHC.

Figure 9. n=4 8-week-old and n=4 21-month-old male C57BL/6J mice were used for nerve fiber quantification. n=3 8-week and n=3 21-month C57BL6/J mice were used for RNAscope, IF and IHC.

Figure S1. 2 male C57BL/6J mice and 1 female C57BL/6J mouse at 8 weeks of age were analyzed by IF and RNAscope.

Figure S2. 2 male C57BL/6J mice and 1 female C57BL/6J mouse at 8 weeks of age were analyzed by RNAscope.

Figure S3. n=2 (1 male and 1 female) Lrig1-CreERT2 R26RnTnG mice were tamoxifen induced at P36 and analyzed at P76.

Figure S4. 1 male and 1 female K14:H2B-GFP mice were analyzed at 8 weeks of age.

Figure S5. 2 male and 1 female C57BL/6J mice were analyzed at 8 weeks of age.

Figure S6. (a-e) n=4 male 8-week and n=4 male 21-month C57BL6/J mice were analyzed. (k) n=4 8-week and n=4 21-month-old male mice were analyzed.

Supplementary Movie 1. 8-week K14:H2B-GFP mice were analyzed; n=2 (1 male and 1 female).

Supplementary Movie 2. Lrig1-CreERT2 ROSAnTnG mice were induced from P40-P42 and analyzed at P102. N=2 males.

| | |
|---|---|
| Wild animals | No wild animals were used in this study. |
| Reporting on sex | Mice of both sexes were analyzed in all experiments except for snRNA-seq, spatial transcriptomics, and aging-related experiments which used only male mice due to limited availability of aged female mice. No notable differences were noted in data from male and female mice. Data are reported disaggregated for sex in the Source Data files. Totals of 141 male mice and 61 female mice were analyzed. |
| Field-collected samples | Not applicable. |
| Ethics oversight | Experiments with KRT14-CreERT2 Smofl/fl mice were carried out under Johns Hopkins University protocol #MO22M104, PI Carlo Iomini; Experiments with KRT14-CreERT Ptch1fl/fl mice were carried out under University of Michigan Unit for Laboratory Animal Medicine protocol #PRO00003165, PI Sunny Wong; experiments with K5-rtTA tetO-GLI2ΔN mice in Figure 5a-c were carried out under University of Pennsylvania IACUC protocol #804809, PI, Sarah E. Millar; all other experiments with mice were carried out under Icahn School of Medicine at Mount Sinai IACUC protocol #2019-0028, PI Sarah E. Millar. |

Note that full information on the approval of the study protocol must also be provided in the manuscript.

## Plants

Seed stocks

*Report on the source of all seed stocks or other plant material used. If applicable, state the seed stock centre and catalogue number. If plant specimens were collected from the field, describe the collection location, date and sampling procedures.*

Novel plant genotypes

*Describe the methods by which all novel plant genotypes were produced. This includes those generated by transgenic approaches, gene editing, chemical/radiation-based mutagenesis and hybridization. For transgenic lines, describe the transformation method, the number of independent lines analyzed and the generation upon which experiments were performed. For gene-edited lines, describe the editor used, the endogenous sequence targeted for editing, the targeting guide RNA sequence (if applicable) and how the editor was applied.*

Authentication

*Describe any authentication procedures for each seed stock used or novel genotype generated. Describe any experiments used to assess the effect of a mutation and, where applicable, how potential secondary effects (e.g. second site T-DNA insertions, mosiacism, off-target gene editing) were examined.*
